# Single-Stage Operation of Hybrid Dark-Photo Fermentation to Enhance Biohydrogen Production through Regulation of System Redox Condition: Evaluation with Real-Field Wastewater

**DOI:** 10.3390/ijms16059540

**Published:** 2015-04-28

**Authors:** Rashmi Chandra, G. N. Nikhil, S. Venkata Mohan

**Affiliations:** 1 Bioengineering and Environmental Sciences (BEES), CSIR-Indian Institute of Chemical Technology (CSIR-IICT), Hyderabad 500 007, India; E-Mails: rashmichandrabhu@gmail.com (R.C.); gnnikhil786@gmail.com (G.N.N.); 2 Academy of Scientific and Innovative Research (AcSIR), CSIR-Indian Institute of Chemical Technology (CSIR-IICT), Hyderabad 500 007, India

**Keywords:** biohydrogen, photosynthetic bacteria, real-field wastewater, dark-photo fermentation

## Abstract

Harnessing hydrogen competently through wastewater treatment using a particular class of biocatalyst is indeed a challenging issue. Therefore, biohydrogen potential of real-field wastewater was evaluated by hybrid fermentative process in a single-stage process. The cumulative hydrogen production (CHP) was observed to be higher with distillery wastewater (271 mL) than with dairy wastewater (248 mL). Besides H_2_ production, the hybrid process was found to be effective in wastewater treatment. The chemical oxygen demand (COD) removal efficiency was found higher in distillery wastewater (56%) than in dairy wastewater (45%). Co-culturing photo-bacterial flora assisted in removal of volatile fatty acids (VFA) wherein 63% in distillery wastewater and 68% in case of dairy wastewater. Voltammograms illustrated dominant reduction current and low cathodic Tafel slopes supported H_2_ production. Overall, the augmented dark-photo fermentation system (ADPFS) showed better performance than the control dark fermentation system (DFS). This kind of holistic approach is explicitly viable for practical scale-up operation.

## 1. Introduction

Recently, a great deal of attention has been paid to the biological production of hydrogen (H_2_) as alternative and eco-friendly fuel throughout the world [1,2]. Microbial conversion of substrate to H_2_ and volatile fatty acid (VFA) by anaerobic fermentation is a complex series of biochemical reactions manifested by diverse group of selective bacteria [3,4]. Accumulation of organic acid metabolites inhibits the H_2_ production process and makes the H_2_ production process unfavorable by limiting the substrate degradation. Further utilization of the organic acids towards H_2_ production is thermodynamically feasible only if there is an additional energy input [5]. This energy input can be in the form of electricity in microbial electrolysis cell (MEC) [6,7,8] or in the form of light in two-stage photofermentation [9,10] or augmentation of photosynthetic bacteria with dark fermentative culture in a single-stage hybrid system [11]. Photofermentataion can be carried out with a wide variety of organic substrates such as carbohydrates; lactate, malate, benzoate and sucrose which are utilized by different species of phototrophic bacteria as electron donors for H_2_ production [12,13,14,15].

Exploitation of wastewater as substrate for H_2_ production with concurrent wastewater treatment is an attractive and effective way of tapping clean energy from renewable resources in a sustainable approach. This provides dual environmental benefits in the direction of wastewater treatment along with sustainable bioenergy (H_2_) generation [16]. Molasses-based distilleries generate 8–15 L of wastewater having high chemical oxygen demand (COD) (100–126 g/L) for every litre of the alcohol produced [17]. Distillery wastewater generated in the form of spent wash or spillage is one of the most complex and strongest industrial organic effluents. It possesses high concentration of biodegradable organic material, such as sugars, lignin, hemicelluloses, dextrin, resins and organic acids [18]. Dairy wastewater contains complex organics, such as polysaccharides, proteins and lipids, which on hydrolysis form sugars, amino acids, and fatty acids [19]. In subsequent acidogenic reaction, these intermediate products are converted to volatile fatty acids (VFA), which are further degraded by acetogens, forming VFA, CO_2_, and H_2_. High organic load and persistent color associated with the distillery and wastewater pose a serious problem to the environment and treatment of such kind of wastewater is challenging [20]. The technologies currently used by distilleries and dairies for treatments of wastewater are biomethanation followed by two-stage biological treatment, concentration and incineration [18]. High organic load, absence of toxic chemicals and availability of large quantities of wastewater may be considered as potential sources for biohydrogen production by integration [21,22]. In this context, a hybrid strategy comprising dark-photofermentation was investigated using designed synthetic wastewater (DSW) as previously reported [11]. In this study, an attempt was made to harvest biohydrogen using real field wastewater (distillery and dairy wastewater obtained from brewery and milk processing industries, respectively) by combining dark and photo-fermentation in a single stage hybrid system using mixed anaerobic bacteria and photosynthetic bacteria.

## 2. Result and Discussion

### 2.1. Bio-Hydrogenesis

#### 2.1.1. Dark-Fermentation (DFS)

**Figure 1:**
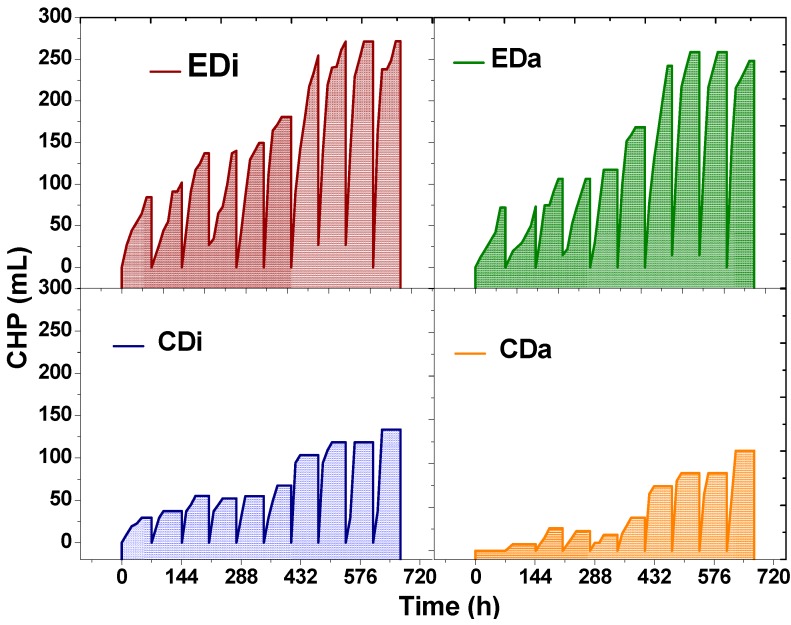
Temporal profile of Cumulative Hydrogen production (CHP) in dark-fermentative system (DFS-CDi, CDa) and augmented dark-photo fermentative system (ADPFS-EDi, EDa) as function of time with dairy and distillery wastewater.

The experimental data depicted feasibility of H_2_ production by utilizing distillery and dairy wastewater as substrate (Figure 1). Acidogenesis of distillery wastewater in control (CDi) resulted in H_2_ production with an initial value of 84 mL during the start-up phase and thereafter gradually increased with cycle operation and reached a maximum consistent value of 133 mL. A similar trend of acidogenesis was observed when the control (CDa) was operated with dairy wastewater. During start-up, the CDa reactor produced 7 mL, and thereafter gradually increased to a maximum of 144 mL. Steady increments in the H_2_ evolution is attributed to the acclimatization and enrichment of H_2_ producing acidogenic bacteria. These experimental results evidenced relatively higher H_2_ production with distillery wastewater than dairy wastewater because of high carbohydrate content in distillery compared with high protein and fat content in dairy waste. Dark-fermentation process involves VFA production as co-metabolites during conversion of organic substrates to H_2_ (Equations (1)–(3)) [3]. The production of VFA affects the buffering capacity that can inhibit the functioning of acidogenic bacteria; perhaps the decline in HPR was consequently noticed.

C_6_H_12_O_6_ + 2H_2_O → 2CH_3_COOH + 2CO_2_ + 4H_2_ (1)

C_6_H_12_O_6_ + 2H_2_O → CH_3_CH_2_CH_2_COOH + 2CO_2_ + 2H_2_ (2)

3C_6_H_12_O_6_ → 4CH_3_CH_2_COOH + 2CH_3_COOH + 2CO_2_ + 2H_2_O (3)

#### 2.1.2. Hybrid Dark-Photo Fermentation

Another set of experiments (EDi and EDa) were carried out where dark fermentation was augmented with photosynthetic bacteria (PSB) (Figure 1). This is designed as hybrid dark-photo fermentation system (ADPFS) operated in a single stage with both the wastewaters, separately. A remarkable performance was noticed with ADPFS relative to controls. Comparing both augmented systems, cumulative H_2_ was noted as higher in EDi (271 mL) than in EDa (248 mL), which is almost double than the controls (CDi, 133 mL and CDa, 114 mL). In the ADPFS, an interesting observation was noticed* i.e.*, the H_2_ production was maximum at the 48th h unlike in the control at 24th h. Higher H_2_ production with ADPFS can be attributed to the co-existence of photosynthetic bacteria with dark-fermentative microflora, which facilitates dark fermentation as well as photofermentataion. The increased duration of H_2_ production is attributed to the role of PSB which are competent in VFA consumption thereby minimizing the acidic stress. The short-chain organic acids (VFA) released during dark-fermentation gets further metabolized to H_2_ by photosynthetic bacteria thereby resulting in higher H_2_ production. Theoretically one mole of acetate can produce four moles of H_2_ while one mole of butyrate can produces 10 moles of H_2_ by photofermentation (Equations (4)–(6)) [3,11]. However, the H_2_ production efficiency and yield was found to depend on the type of wastewater and amount of organic content which is biodegradable.

2CH_3_COOH + Light→ 2CO_2_ + 4H_2_ (4)

CH_3_CH_2_CH_2_COOH + 3H_2_O + Light → 4CO_2_ + 10H_2_ (5)

HCOOH + Light → CO_2_ + H_2_ (6)

### 2.2. Pigments and Biomass

**Figure 2:**
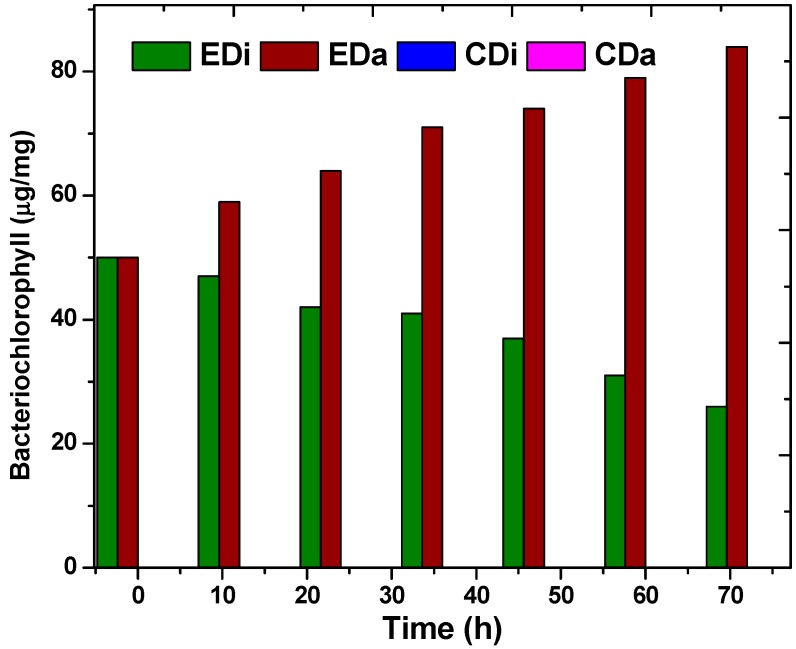
Pigment analysis (bacteriochlorophyll) of dark-fermentative system (DFS-CDi, CDa) and augmented dark-photo fermentative system (ADPFS-EDi, EDa) as function of time with distillery and dairy wastewater.

**Figure 3:**
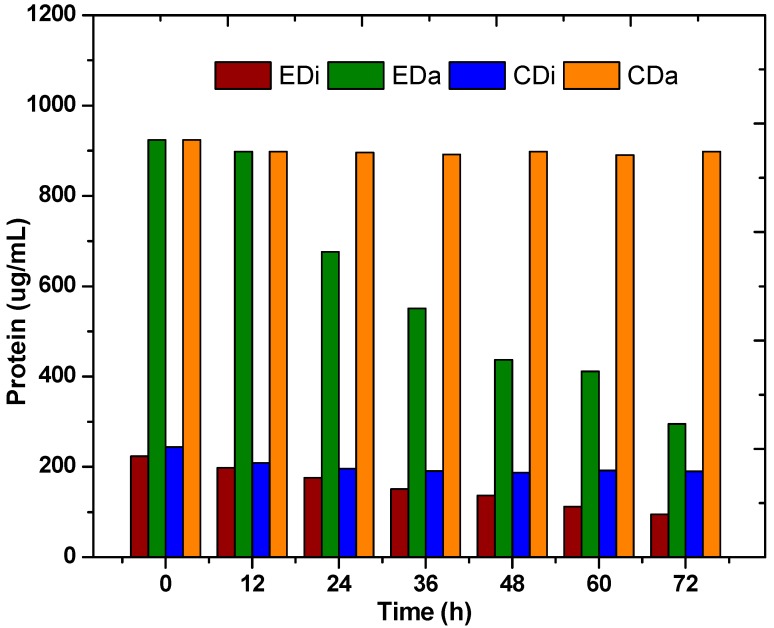
Variation in protein content in dark-fermentative system (DFS-CDi, CDa) and augmented dark-photo fermentative system (ADPFS-EDi, EDa) as a function of time with distillery and dairy wastewater.

**Figure 4:**
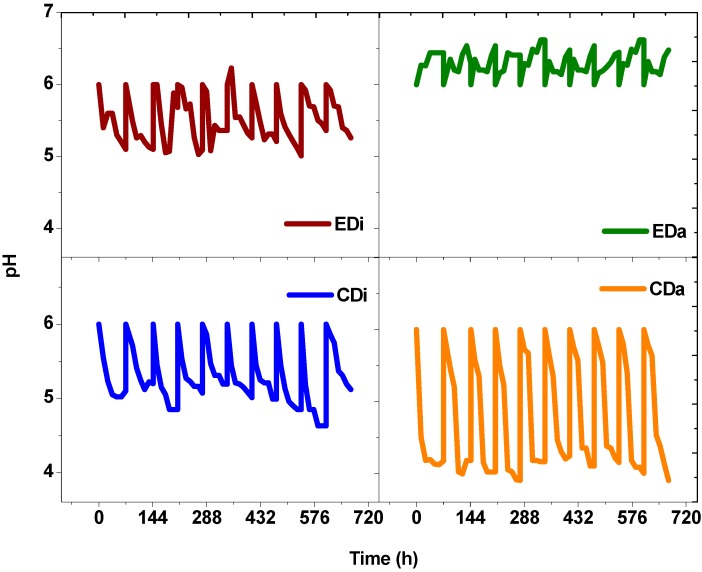
Changes in pH profile as function of time with dairy and distillery wastewater in dark-fermentative system (DFS-CDi, CDa) and augmented dark-photo fermentative system (ADPFS-EDi, EDa).

Biomass concentration of photosynthetic bacteria was calculated indirectly via bacteriochlorophyll estimation. Bacteriochlorophyll (*BChl*) is a pyrrole derivative specific to photosynthetic bacteria and plays a major role in anoxygenic photosynthesis [23]. To confirm the growth of photosynthetic bacteria during ADPFS operation of all experimental variants, *BChl* was analyzed (Figure 2). Control systems (CDi and CDa) did not contain PSB throughout the study. On the other hand, augmented systems (EDi and EDa) showed growth of PSB. Among the two experimental runs, EDa showed significant increments in photosynthetic *BChl* over a cyclic operation and a maximum of 84 µg/mg at 72 h was noticed. Dairy wastewater is rich in protein and is a good source of nitrogen for the PSB biomass growth (Figure 3). However, in the case of the EDi system, a decline in PSB growth was noticed at the end of each cycle. Therefore, prior to the start of each batch, a fixed inoculum volume of 10 mL was added to the ADPFS and at the end of the batch 26 µg/mg (72 h) was noted. The deficiency in PSB growth is possibly attributed to the absence of protein in distillery wastewater. Besides, the decrease in PSB biomass is attributed to the acidic shock caused by VFA present in the system. *BChl* is pH sensitive and at acidic pH structural and functional aspects of *BChl* to *PhBChl* was previously reported [11]. In the present experiment, the pH drop and VFA present in the system was sufficient to trigger pheophytinization of the *Bchl* which automatically hinders bacterial photosynthetic activity. Pheophytinization is a bio-physio-chemical process where at low pH and high proton (H^+^) concentration, the central metal ion (Mg^2+^) of *BChl* gets bleached out and is replaced by H^+^ ion [11,24,25,26]. Decrease in pH was noticed in DFS (CDi and CDa) due to anaerobic fermentation and release of VFA. But, in case of hybrid ADPFS (EDi and EDa) the pH increase was noticed due to the consumption of VFA (Figure 4). In this regard, to revive the batch, an aliquot of PSB inoculum was added to the system.

### 2.3. Total Volatile Fatty Acids and Composition

**Figure 5:**
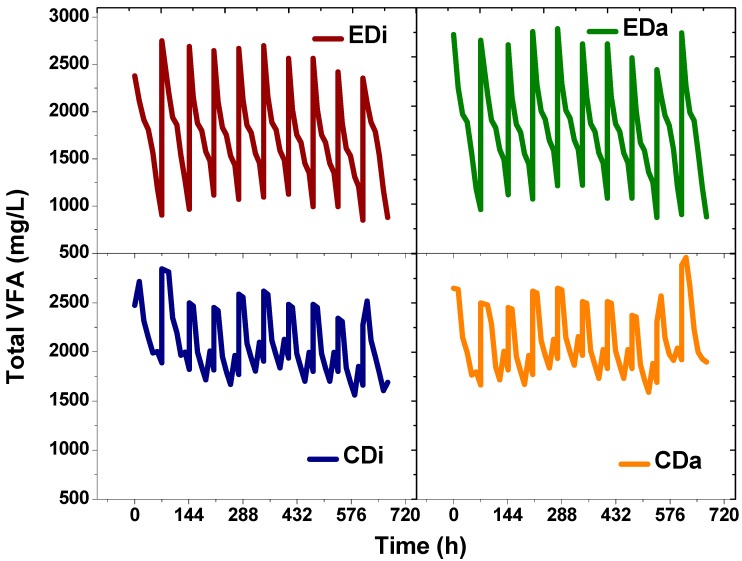
Total volatile fatty acids (VFA) profile in dark-fermentative system (DFS-CDi, CDa) and augmented dark-photo fermentative system (ADPFS-EDi, EDa) as a function of time with dairy and distillery wastewater.

**Figure 6:**
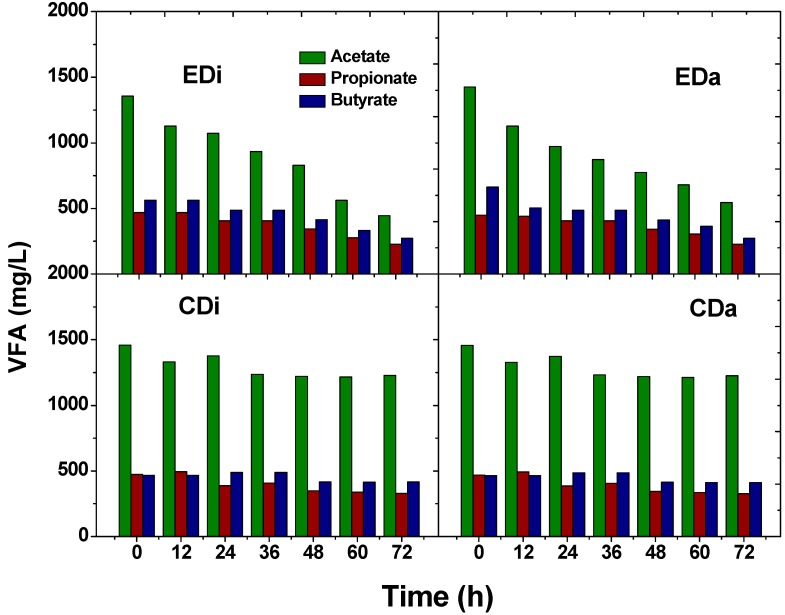
Compositional analysis of volatile fatty acids composition as a function of time with distillery and dairy wastewater in dark-fermentative system (DFS-CDi, CDa) and augmented dark-photo fermentative system (ADPFS-EDi, EDa).

Both the wastewaters contained a certain amount of VFA prior to the start-up of experiments; distillery wastewater (2500 ± 200 mg/L) and dairy wastewater (2744 ± 200 mg/L). Thereafter, when fed to control systems (CDi and CDi) and ADPFS (EDi and EDa) changes in total VFA concentration and its composition were observed. In CDi operation, VFA concentration increased to a maximum value of 2519 ± 200 mg/L at 12 h and thereafter decreased to 1690 mg/L at the end of the batch. Similarly, in CDa, VFA increased to a maximum value of 2962 mg/L at 12 h and thereafter declined to 1898 mg/L at the end of the batch. These variations were much seen in case of EDi and EDa where the initial/final total VFA concentrations were 2356/880 and 2744/872 mg/L, respectively (Figure 5). VFA production is associated with conversion of organic fraction to acid intermediates in the anaerobic microenvironment. Change in the concentration of acid metabolites can affect the system buffering capacity and higher acid concentrations will inhibit the function of acidogenic bacteria specifically, H_2_ production. Therefore, individual composition was also analyzed using HPLC which assists in tracking the biochemical pathway of the biocatalyst during process operation. Acetate or butyrate pathway favors H_2_ production, while propionic acid is not as favorable for H_2_ production [27]. In the present study, a higher proportion of acetic acid along with butyric acid and propionic acid was observed in the controls (CDi and CDa). Initial concentrations of observed VFA (CDi-acetate, 1457 mg/L; propionate, 469 mg/L; butyrate, 463 mg/L; CDa-acetate, 1459 mg/L; propionate, 472 mg/L; butyrate, 466 mg/L) were changed by the end of the batch (CDi-acetate, 1225 mg/L; propionate, 327 mg/L; butyrate, 412 mg/L; CDa-acetate, 1228 mg/L; propionate, 330 mg/L; butyrate, 415 mg/L) (Figure 6). The temporal profile of these acids did not show much variation indicating the inefficiency of the dark-fermentative consortia. However, a remarkable change in the acid composition profile was noticed in the same wastewaters when fed to the experimental set-up inoculated with PSB The initial concentrations of acetic, butyric and propionic were (EDi-acetate, 1357 mg/L; propionate, 469 mg/L; butyrate, 563 mg/L; EDa-acetate, 1427 mg/L; propionate, 449 mg/L; butyrate, 663 mg/L) decreased by the end of the batch (EDi-acetate, 445 mg/L; propionate, 227 mg/L; butyrate, 272 mg/L; CDa-acetate, 545 mg/L; propionate, 227 mg/L; butyrate, 272 mg/L). VFA removal was about 62% in the EDi system and 68% in the EDa system. Remarkably, total VFA in ADPFS removed about 50% less than control DFS. The above experiment (ADPFS) documented the functional role of PSB in utilizing the VFA for its growth and maintenance. Interestingly, dairy wastewater showed higher removal of VFA in spite of less H_2_ production which was due to the utilization of these VFAs towards the biomass production. Besides, dairy wastewater contains high protein content which is a good nitrogen source for PSB growth. Although VFA removal was observed in distillery wastewater, PSB growth declined with batch time and also the protein content was minimal, which supports maintenance but not growth [3,11].

### 2.4. Substrate Degradation

**Figure 7:**
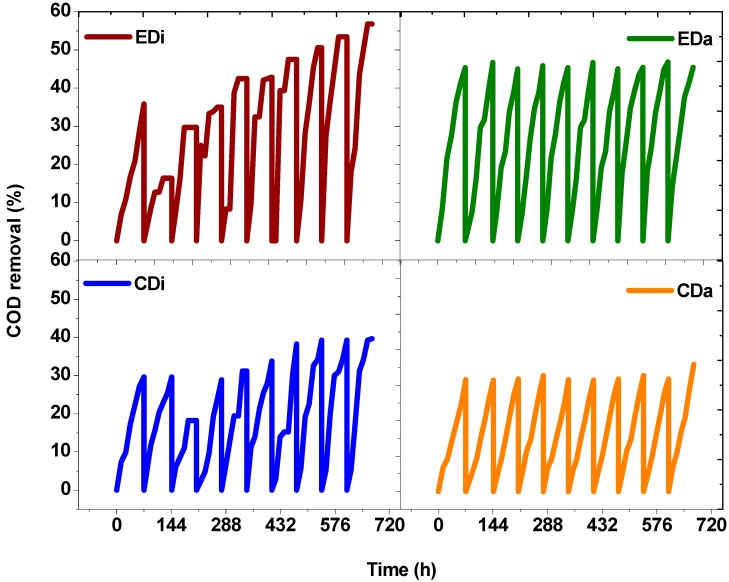
Substrate utilization in terms of chemical oxygen demand (COD) removal as function of time with distillery and dairy wastewater in dark-fermentative system (DFS-CDi, CDa) and augmented dark-photo fermentative system (ADPFS-EDi, EDa).

Substrate degradation efficiencies (based on COD) showed variation as a function of biocatalyst and complexity of wastewater. EDi operation showed COD removal efficiency of 18% at the 12th h which improved with time (24 h-24.32%, 36 h-43.52%; 48 h-50.80%; 60 h-56.80%) and approached a maximum value of 56.8% at the 72nd h. EDa operation showed COD removal efficiency of 14% at the 12th h which improved with time (24 h-21.72%, 36 h-29.81%; 48 h-37.69%; 60 h-41.31%) and approached a maximum value of 45.42% at 72nd h. CDi operation showed COD removal efficiency of 5.36% at the 12th h which improved with time (24 h-17.31%, 36 h-31.20%; 48 h-34.69%; 60 h-39.63%) and approached a maximum value of 39.63% at 72nd h. CDa operation showed COD removal efficiency of 6.89% at the 12th h which improved with time (24 h-10.85%, 36 h-16.84%; 48 h-20.84%; 60 h-28.84%) and approached a maximum value of 35.42% at the 72nd h (Figure 7). Almost 20% increment in substrate degradation efficiency was observed with augmented operation which also demonstrated the effective functioning of photosynthetic consortia in treating wastewater. CDi and CDa operation showed comparatively lower substrate degradation. Microbial fermentation generates energy-rich reducing power (NADH,* etc.*), which subsequently gets re-oxidized during respiration with simultaneous generation of biological energy molecules (ATP) in the presence of a terminal electron acceptor (TEA). Anaerobic respiration has the ability to utilize a wide range of organic compounds by the acidogenic pathway and generates VFA in association with H_2_. Hydrogenase plays an important role for the generation of H_2_. Under anaerobic conditions, photosynthetic bacteria use sunlight as a source of energy and produce H_2_ and CO_2_ by degrading organic molecules [1]. The observed higher H_2_ production in hybrid system might be attributed to consumption of VFA by photosynthetic consortia towards additional H_2_. Light absorption by bacteriochlorophyll (BChl) molecules initiates e^−^ transfer from a reaction center to quinine pool (QA) and then to the cytochrome subunit for generating a H^+^ gradient, which finally gets reduced to H_2_. The ability of PSB to trap energy over a wide range of the light spectrum without producing oxygen and its versatility in utilizing various substrates like acetate, butyrate and propionate makes the hybridization of photo fermentation with dark fermentation a feasible and viable approach [2,11,28,29].

### 2.5. Bio-Electrocatalytic Analysis

**Figure 8:**
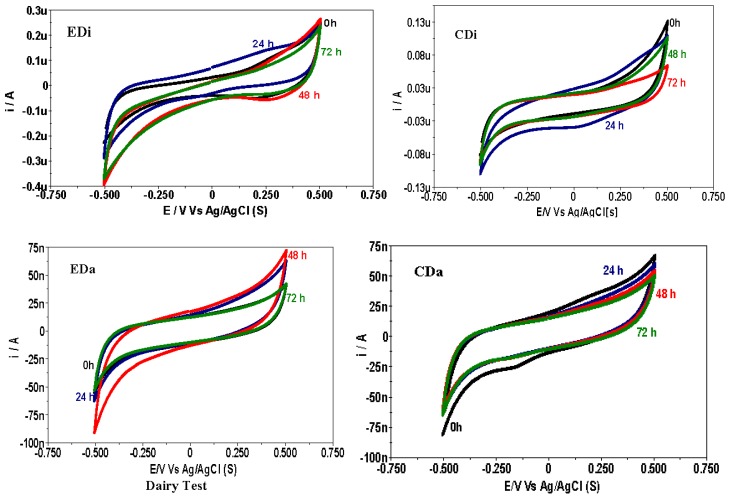
Cyclic voltammograms of dark-fermentative system (DFS- CDi, CDa) and augmented dark-photo fermentative system (ADPFS- EDi, EDa) operated with distillery and dairy wastewater.

Electron-transfer reactions are integral components in every act of microbial metabolism. These electron discharges are studied using bio-electrochemical techniques viz., cyclic voltammetry (CV). This is a highly versatile technique which allows understanding of the electron transfer from redox mediator species of the biocatalyst into solution using a three electrode set-up. Understanding the behavior of biocatalyst and its nature by scanning a definite potential window during H_2_ production is a significant aspect of this study. Voltammogram profiles (*vs.* Ag/AgCl) showed significant variation in both control (DFS) and experimental (ADPFS) set-up with both the wastewaters. Reduction current (RC) was relatively higher compared to the oxidation current (OC) in ADPFS operation, indicating a feasible anaerobic microenvironment for the evolution of hydrogen (Table 1). Analogous observations relating CHP was noticed in ADPFS (EDi and EDa) which was previously discussed. In the case of control DFS (CDi and CDa) as well the RC was higher than OC, and similarly, H_2_ production was relatively higher in distillery wastewater than dairy wastewater. The catalytic redox currents varied with batch time and the peak values were corroborating RC with maximum CHP and/or OC with substrate degradation. In CDi, the RC gradually increased from the 0th h (0.07 µA) and increased to a peak value at 24th h (0.10 µA) and thereafter decreased at 72nd (0.06 µA). In CDa, RC decreased along the batch process (0.081 µA at 0th and 0.064 µA at 72nd h) which is not favorable for H_2_ production. Likewise in case of EDi, the RC gradually increased from the 0th h (0.23 µA) to a peak value at the 48th h (0.40 µA) and thereafter declined at the 72nd h (0.37 µA). The disparity between CDi and EDi is the presence of PSB that involves acid consumption and consequently results in higher H_2_ than DFS. While, EDa responded in the same manner as in case of CDa,* i.e.*, the RC values decreased from the batch start-up (0th h, 0.09 µA) till the end of the batch (72nd h, 0.053 µA) (Figure 8).

**Table 1:** Comprehensive results of bioelectrochemical analysis carried out for both the control (DFS) and experimental systems (ADPFS).

	Time (h)	OC (µA)	RC (µA)	β_c_ (V/dec)	β_a_ (V/dec)	*R*_p_ (kΩ)
**EDi**	0	0.25	0.23	0.219	0.408	15,216
	24	0.24	0.29	0.263	0.625	11,473
	48	0.27	0.40	0.157	0.352	14,734
	72	0.24	0.37	0.165	0.377	15,173
**CDi**	0	0.12	0.07	0.257	0.544	15,038
	24	0.10	0.10	0.121	0.557	16,325
	48	0.08	0.08	0.059	0.667	15,440
	72	0.06	0.06	0.070	0.632	15,290
**EDa**	0	0.07	0.09	0.683	0.158	16,430
	24	0.06	0.06	0.205	0.622	17,580
	48	0.041	0.055	0.165	0.607	12,300
	72	0.042	0.053	0.155	0.726	16,480
**CDa**	0	0.067	0.081	1.770	0.576	13,330
	24	0.060	0.064	0.212	0.584	18,890
	48	0.054	0.060	0.192	0.608	18,136
	72	0.050	0.064	0.184	0.681	17,020

DFS, dark fermentation system; ADPFS, augmented dark photo fermentation system; OC, oxidation current; RC, reduction current; β_c_, reduction slope; β_a_, oxidation slope; *R*_p_, polarization resistance.

Further, these CV were analyzed for Tafel slopes which reveal the bioelectro-kinetic behavior of the biocatalyst in terms of exchange current density and electron transfer coefficients (oxidative β_a_, reductive β_c_). The electron transfer during redox reactions between the biocatalyst and solid electrode need to overcome different barriers referred to as overpotentials. Higher oxidation slope suggests the requirement of higher activation energy that makes oxidation less favorable and vice versa. The same relationship applies to the reduction slope. Remarkable variation was seen in these slope values both with batch time and operating process condition (specifically nature of biocatalyst and wastewater used). However, overall the β_c_ values were lower than the β_a_ values which supports for a reductive microenvironment which is congenial for H_2_ production. Rate of change in β_c_ indicated the bioelectro-kinetics of biocatalyst used and variable profiles were noted with control and experimental systems. Interestingly, this change corroborated the RC of CV and CHP at that particular batch time. Besides, β_c_ values were lower in control than in ADPFS, which indicates that dark-fermentative consortia have more tenacity for H_2_ production. But, this was not observed so in this study because of the VFA accumulation, which might have disturbed the biocatalyst buffer capacity leading to a drop in pH. On the other hand, in ADPFS the VFA are utilized in the process of H_2_ production.

Besides, polarization resistance (*R*_p_) refers to the electron transfer from the biocatalyst at the solution electrode interface which is derived from Tafel analysis. The higher the *R*_p_, the lower will be the electron transfer making the process less favorable. In this study, the *R*_p_ values are comparatively on a higher side because the electrode assembly is not continuously put in the system which assists in biofilm formation and for better electron conduction. The redox catalytic currents measured are due to suspended biocatalyst in the electrolyte, and in this manner heavy electron losses and resistances (referred to overpotentials) are noticed. The Tafel analysis therefore reports high activation loss (referred to as *R*_p_). However, the solution conductivity is a criterion which showed considerable variation with the nature of wastewater used and the biocatalyst composition. In this study, *R*_p_ values were relatively lower with distillery wastewater than dairy wastewater and besides, these values were higher in control DFS than experimental ADPFS (Table 1). Notably the polarization resistance affects the Hydrogen Evolution Reaction (HER) kinetics which is consecutively influenced by the microbial catalytic capability within the tested potential region [30]. Perhaps, this could be a possible reason for high RC response and correspondingly higher H_2_ production.

## 3. Experimental Section

### 3.1. Biocatalyst

#### 3.1.1. Anaerobic Culture

Anaerobic consortia acquired from a full scale operating anaerobic treatment unit was used as dark fermentative inoculums in the experiments. It was initially enriched in designed synthetic wastewater (DSW) (0.5 g/L NH_4_Cl, 0.25 g/L KH_2_PO_4_, 0.25 g/L K_2_HPO_4_, 0.3 g/L MgCl_2_∙6H_2_O, 0.025 g/L FeCl_3_, 0.016 g/L NiCl_4_, 0.025 g/L CoCl_2_, 0.0115 g/L ZnCl_2_, 0.0115 g/L CuCl_2_, 0.005 g/L CaCl_2_, 0.015 g/L MnCl_2_, 3.00 g/L C_6_H_12_O_6_) for a period of 72 h comprising 3 cycles each with 24 h under anaerobic microenvironment at pH 6.0 (100 rpm; 48 h). After enrichment of the inoculum it was subjected to sequential pretreatment with chemical, heat-shock and acid-shock to enrich H_2_ producers (hydrogenic bacteria) as well as to suppress methanogenic bacteria (MB) [31,32].

#### 3.1.2. Photosynthetic Culture

An indigenous mixed photosynthetic consortium was acquired from existing photosynthetic hydrogen producing system reported in our previous experiments [11]. This culture was enriched in a succinate salt broth, consisting of 0.33 g KH_2_PO_4_, 0.33 g MgSO_4_∙7H_2_O, 0.33 g NaCl, 0.5 g NH_4_Cl, 0.5 g CaCl_2_∙2H_2_O, 1.0 g sodium succinate, 0.02 g yeast extract, 1 L Distilled H_2_O, 1 mL trace metal solution (10 mg ZnSO_4_∙7H_2_O, 3 mg MnCl_2_∙4H_2_O, 30 mg H_3_BO_3_, 20 mg CoCl_2_∙6H_2_O, 1 mg CuCl_2_ 2H_2_O, 2 mg NiCl_2_∙6H_2_O, 3 mg Na_2_MoO_4_, 1.0 L Distilled H_2_O, pH 7) and 0.5 mL 0.02% FeSO_4_∙7H_2_O solution. This composition works well for enrichment of photosynthetic bacteria from natural sources. The cells were anaerobically grown in an incubator provided with an illumination (4000 lux (10.2 W/m^2^); 30 °C) for 7 days to reach logarithmic growth phase.

### 3.2. Wastewater

#### 3.2.1. Dairy Wastewater

Dairy wastewater (suspended solids (SS), 1590 mg/L; total dissolved solids (TDS), 8840 mg/L; total solids (TS), 10,430 mg/L; COD, 11 g/L; BOD, 7173 mg/L, pH 7.2; nitrates, 1943 mg/L; phosphate, 22 mg/L; sulphate, 101 mg/L) was used as substrate. The wastewater can be considered as complex in nature (BOD/COD ≈ 0.61) due to the presence of proteins, carbohydrates, and fat content. After collection, the wastewater was transferred immediately to the laboratory and stored at 4 °C. Wastewater was diluted using tap water to requisite organic loading rate (OLR) prior to feeding and pH adjustment.

#### 3.2.2. Distillery Wastewater

Distillery wastewater (Suspended solids (SS), 13,500 mg/L; total dissolved solids (TDS), 11,600 mg/L; total solids (TS), 25 g/L; COD, 124 g/L; BOD, 35 g/L, pH 8.2, nitrates, 1943 mg/L; phosphate, 80 mg/L; sulphate, 92 mg/L; alkalinity, 4000 mg/L and chlorides, 30 mg/L) was used as substrate. The wastewater can be considered as complex in nature (BOD/COD ~ 0.29) due to the presence of proteins, carbohydrates, and lipids content. After collection, the wastewater was transferred immediately to the laboratory and stored at 4 °C. Wastewater was diluted using tap water to requisite organic loading rate (OLR) prior to feeding and pH adjustment.

### 3.3. Experimental Methodology

**Figure 9:**
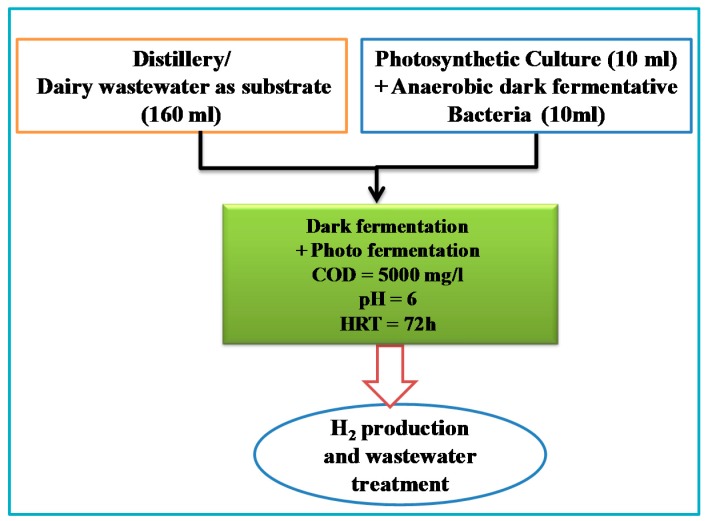
Schematic representation of experimental set-up employed.

Two set of experiments were carried out separately with distillery and dairy wastewater to investigate the augmentation effect of photosynthetic bacteria over H_2_ production (Figure 9). Control is a dark fermentative system (DFS) which is operated only with dark anaerobic mixed bacterial culture and designated as control distillery-CDi and control dairy-CDa. On the other hand, experimental systems are augmented dark-photo fermentative system (ADPFS) which is a hybrid setup operated with mixed photosynthetic bacteria (PSB) and designated as experimental distillery-EDi and experimental dairy EDa. All the experiments were operated in batch mode with a total/working volume of 250/180 mL (160 mL wastewater + 20 mL inocula) under anaerobic conditions. In the control, only dark anaerobic culture is taken; while, in the experimental system 10 mL dark and 10 mL PSB are taken for study. CDi and CDa experimental setups were wrapped with aluminum foil to restrict the penetration of light, whereas EDi and EDa ware illuminated with LED lights at intensity of 4000 lux (10.2 W/m^2^). All the systems were subjected to continuous agitation (120 rpm) using a temperature-controlled orbital shaker at an ambient temperature of 30 °C. Retention time of the batch experiments was kept as 72 h. The pH of wastewater was adjusted to 6, prior to inoculating the culture. All experiments were operated at organic loading rates (OLR) of 1.0 kg COD/m^3^-day. Organic loads (OL) were decided based on our previous experiments [11].

### 3.4. Analytical Methods

Production of H_2_ was estimated using a microprocessor based electrochemical gas sensor (ATMI GmBH Inc., Unterhaching, Germany). The output signal displayed represents the percentage volume of H_2_ accumulated in the headspace of the septum flasks. The variations in the bioprocess and microenvironment of both systems during H_2_ production was assessed by monitoring the COD removal efficiency, pH change during the operation and volatile fatty acid (VFA) profiling as per the standard methods [33]. Organic loading rate (kg COD/m^3^-day) based on COD and substrate degradation rate (SDR-kg COD_R_/m^3^-day) was calculated to study the rate and pattern of COD removal during the operation. VFA profile was monitored using high performance liquid chromatography (HPLC; UV detector; C18 reverse phase; 5 m particle size; flow rate-0.6 mL/h; wavelength-210 nm; mobile phase-acetonitrile (40%) in 1 mN H_2_SO_4_). At the end of each cycle total Bacteriochlorophyll (Bchl) was estimated by colorimetric procedure by centrifuging 5 mL of the bacterial culture (10,000 rpm for 10 min) and the supernatant was discarded. The pellet was re-suspended in 0.1 mL of milli-Q water to which 4.9 mL methanol was added to extract the pigments. Again, the culture was re-centrifuged to sediment the extracted cells and the supernatant were used for pigment analysis. The extinction coefficient of BChl of the extract was determined at 775 nm in a spectrophotometer and the BChl content was calculated by multiplying OD by a multiplication factor 2.19 [23,24]. Bio-electrocatalytic behavior of all the experimental variants was studied* in situ* by using a potentiostat-galvanostat system (Autolab-PGSTAT12, Utrecht, The Netherlands). Voltammograms (cyclic) were recorded by applying a potential ramp at a scan rate of 30 mV/s over the range from +0.5 to −0.5 V. A scan rate of 30 mV/s was optimized for the system, which showed significant interfacial electron-transfer kinetics. All the electrochemical assays in DFS and ADPFS were performed considering graphite (anode) as working electrode (WE) and platinum (cathode) as counter electrode (CE) against Ag/AgCl (s) reference electrode (RE) [29].

## 4. Conclusions

This study demonstrated the feasibility of biological H_2_ generation from distillery and dairy wastewater treatment in a single stage hybrid system. The hybrid system comprising dark and photo-fermentation facilitated higher H_2_ production with distillery wastewater over dairy waste because of its high organic fractions compared with dairy waste, which was protein-rich. An increment of about 40% was noticed in H_2_ production in photo-augmented system with simultaneous reduction in accumulated VFA. This study interprets the performance of hybrid process in a single system which is likely to provide the crucial information for the development of a full scale integrated photo-fermentation system.
